# An assessment of information needs and workflows for emergency service providers and caregivers of children with medical complexity

**DOI:** 10.1186/s12913-023-09366-y

**Published:** 2023-05-08

**Authors:** Aubree Honcoop, Russell J. McCulloh, Ellen Kerns, Bethany Lowndes, Tiffany Simon, Natalie McCawley, Ricky Flores, Martina Clarke

**Affiliations:** 1 University of Nebraska Medical Center, 42nd and Emile St, Omaha, NE USA; 2 Children’s Hospital and Medical Center, Omaha, NE USA; 3 University of Nebraska Omaha, Omaha, NE USA

**Keywords:** Emergency Information Form, Children with medical complexity, Needs Assessment

## Abstract

**Background:**

The goal of Project Austin, an initiative to improve emergency care for rural children who are medically complex (CMC), is to provide an Emergency Information Form (EIF) to their parents/caregivers, to local Emergency Medical Services, and Emergency Departments. EIFs are standard forms recommended by the American Academy of Pediatrics that provide pre-planned rapid response instructions, including medical conditions, medications, and care recommendations, for emergency providers. Our objective is to describe the workflows and perceived utility of the provided emergency information forms (EIFs) in the acute medical management of CMC.

**Methods:**

We sampled from two key stakeholder groups in the acute management of CMC: four focus groups with emergency medical providers from rural and urban settings and eight key informant interviews with parents/caregivers enrolled in an emergency medical management program for CMC. Transcripts were thematically analyzed in NVivo© by two coders using a content analysis approach. The thematic codes were combined into a codebook and revised the themes present through combining relevant themes and developing of sub-themes until they reached consensus.

**Results:**

All parents/caregivers interviewed were enrolled in Project Austin and had an EIF. Emergency medical providers and parents/caregivers supported the usage of EIFs for CMC. Parents/caregivers also felt EIFs made emergency medical providers more prepared for their child. Providers identified that EIFs helped provide individualized care, however they were not confident the data was current and so felt unsure they could rely on the recommendations on the EIF.

**Conclusion:**

EIFs are an easy way to engage parents, caregivers, and emergency medical providers about the specifics of a care for CMC during an emergency. Timely updates and electronic access to EIFs could improve their value for medical providers.

**Supplementary Information:**

The online version contains supplementary material available at 10.1186/s12913-023-09366-y.

## Background

Children with medical complexity (CMC) are one of the fastest-growing segments of the pediatric population, representing 12% of emergency department visits and up to 40% of pediatric hospitalizations in the Medicaid system annually [1–3]. Medical complexity includes conditions that depend on technology for daily life (e.g. feeding tubes), congenital defects, and acquired multisystem diseases that require complex medication regimens [4]. When CMC present to emergency medical providers with an acute illness, these providers need to quickly comprehend the child’s unique situation to initiate care [5, 6]. A lack of accurate and concise information in critical situations creates the opportunity for knowledge gaps, exposing CMC to the possible risk of injury or death through delayed recognition of life-threatening illness or initiation of inappropriate (and potentially harmful) medical interventions [7–10]. Consequently, identifying ways to improve emergency care of CMC could have profound consequences on healthcare resource utilization locally and nationally.

The Coordinating All Resources Effectively (CARE) Award, funded by the Centers for Medicare & Medicaid Innovation, was designed to improve coordination of care for 8,000 enrolled CMC and their families through 10 children’s hospitals [11]. Since 2016, Children’s Hospital and Medical Center in Omaha, NE has conducted a program that creates Emergency Information Forms (EIFs) for families and emergency medical providers in tandem with targeted education and outreach to healthcare providers across our region. This program, named Project Austin, has enrolled over 1,200 patients and 400 healthcare organizations [12]. The emergency medical providers that commonly work with Project Austin include Emergency Medical Services (EMS) staff, ambulance teams, and Emergency Departments (EDs). Project Austin is designed to assist with information needs during critical situations on site by EMS, transport management by ambulance teams, and in local EDs.

EIFs for CMC are recommended by the American Academy of Pediatrics (AAP) and American Academy of Emergency Physicians [7]. An EIF is a standardized sheet of paper containing a concise medical summary of a child’s medical conditions, medications, and special health care needs designed to inform emergency medical providers so they can quickly provide the best possible care. Parents/caregivers often are not reliable sources of information during acute medical events, and so these forms have been shown to improve emergency provider confidence and reduce patient complications in simulation scenarios and in two EDs in tertiary care children’s centers [13–15]. Project Austin provides these forms to families that are part of the Complex Care Clinic at Children’s Hospital and Medical Center (CHMC) in Omaha, NE, and makes those forms available both physically locally to EMS and ED personnel and virtually through the Electronic Health Record (EHR). These bright orange EIFs are recommended to families to be kept up to date at home, schools, childcare centers, in vehicles, and with their child’s belongings in case of crisis. Project Austin families are instructed to inform emergency medical providers of their child’s participation in the program. Emergency medical providers, both EMS and ED staff, throughout the state of Nebraska receive training and education regarding Project Austin, the use of EIFs, and on medical topics relevant to the acute management of CMC.

Project Austin provides an excellent opportunity to identify information needs encountered by families and healthcare providers when caring for CMC during a medical emergency, from the onsite EMS teams to arrival to the ED. Knowledge gaps exist, however, about how families, emergency medical services, and emergency departments utilize the EIF as part of Project Austin and how useful emergency medical providers find the information provided within the forms. Additional gaps exist regarding the optimal management and maintenance of the paper forms across various emergency medical service providers, as well as what makes providers confident of the accuracy of the information contained within the EIFs during a medical emergency. The objective of the present study is to describe the information needs of emergency medical providers and family parents/caregivers enrolled in Project Austin when managing CMC facing acute illness.

## Methods

### Research Team and Reflexivity

#### Personal characteristics

Focus groups were conducted by authors BL and MC. BL has an MPH (Master of Public Health) and a PhD in Biomedical Engineering, and she is an assistant professor in the Department of Neurological Sciences at the University of Nebraska Medical Center (UNMC). MC has a PhD in Health Informatics, and she is an assistant professor in the School of Interdisciplinary Informatics at the University of Nebraska Omaha. Both BL and MC have experience leading focus groups. Key informant interviews were conducted by author AH. They are an MD/PhD graduate student at UNMC and were trained to conduct interviews by BL and MC.

#### Relationship with participants

Research participants for both the focus groups and key informant interviews have not previously been contacted by the researchers, but did have a pre-existing relationship with authors RM, TS, and NC through the Complex Care Clinic at CHMC and Project Austin. Focus groups and key informants were told the interviewers’ names and occupations, as well as the research study’s goals and future directions.

### Study Design

#### Theoretical Framework

A content analysis approach was used to code narrative themes. The interview guide for focus groups and key informant interviews (Appendix 1) had separate goals. Focus groups with healthcare professionals sought to identify critical information and workflows surrounding EIFs in use during a critical situation both at home and in the ED. Key informant interviews with parents/caregivers of CMC identified participants who were a part of Project Austin to discuss the creation of the EIF and the process of Project Austin activation. Members of the research team acting as secondary leads and taking field notes asked relevant follow-up questions based on participants’ responses. questions from each are in Table 1.

**Table 1:** Example Questions from Interview Guides

Focus Group Questions	What information is critical to providing emergency care to medically complex kids? How do you gather this information? Describe the process of retrieving information from the EIF. Was information missing? Were you able to change the way you provided care based on the information?
Key Informant Questions	Is your child a part of Project Austin? Does your child have an EIF? Describe to me the process of creating the EIF for your child. Have you had to activate Project Austin and access the EIF in an emergency situation? What went well during that situation? What could have gone better?

#### Participant selection

Participants were a convenience sample recruited across the catchment area for Project Austin. Hospitals were contacted in two rural and two urban locations for focus group recruitment. Focus groups were advertised through fliers at all sites - Broken Bow, Columbus, Omaha, and Lincoln, NE - and department email servers in Omaha and Lincoln, NE. A sample of providers and EMS were offered a chance to participate in these focus groups. Emergency medicine providers eligible if their job title made them a part of emergency department staff – physicians, nurses, and administrative personnel – or emergency medical service (EMS) staff – paramedics, ambulance teams, and local fire department members. Key informant interviewees were parents and caregivers who presented to the Complex Care Clinic at CHMC within the study period. The Complex Care Clinic at CHMC is for children with three or more complex conditions, including a variety of developmental disabilities, multiple congenital anomalies, chromosomal abnormalities, and children who require visits to many different specialists [16]. Participants were approached and consented to participate through emails, phone calls, and face-to-face, as allowed by the SARS-CoV-2 pandemic. This study was approved by the University of Nebraska Medical Center Institutional Review Board and all research was carried out according to relevant guidelines and regulations.

#### Setting

Focus groups from rural areas were done in person and focus groups from urban areas were conducted virtually. Focus groups consisted of a lead and a secondary moderator taking field notes. All key informant interviews were virtual because of city and hospital lockdown due to the COVID-19 pandemic. Key informant interviews only had a lead interviewer. Broken Bow and Columbus, NE were considered rural areas, and Lincoln and Omaha, NE were urban areas. Study period was from December 2019 to December 2020.

### Data Collection

The interview guide provided questions as prompts for semi-structured discussion for both the focus groups and the key informant interviews. No repeat interviews were conducted. All focus groups and key informant interviews were audio recorded to ensure completeness in the data collection. The focus groups lasted less than one hour, and the key informant interviews were less than 30 min. Sampling continued until thematic saturation was reached, meaning that no added information was gained during the interviews [17]. Transcripts were not returned to the participants for comment or correction.

### Analysis and findings

Concurrent data collection analysis was conducted through various stages of coding for the focus groups; analysis for key informant interviews was completed after all data was collected. Audio files were de-identified and transcribed, and two independent, qualitative coders (AH, MC) analyzed the transcribed interviews, with a third coder (RM) serving as a tiebreaker in situations of disagreement. A codebook was created and organized based on themes found through data analysis, and themes were revised until coders came to a consensus. Qualitative data was analyzed using NVivo©, a qualitative data analysis software (QSR International, Doncaster, Australia). Participants did not provide feedback on the findings.

## Results

### Participants

**Fig. 1:**
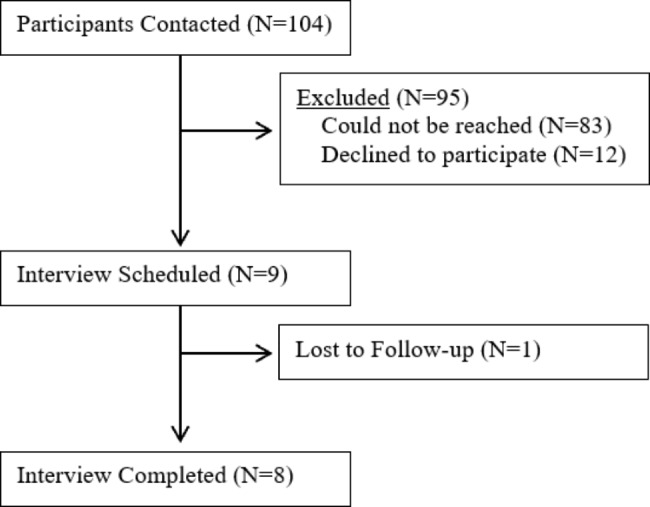
Key Informant Interview Recruitment Flow Diagram. This diagram represents the number of parents and caregivers identified as eligible and approached to participate through the complex care clinic at CHMC in Omaha

In total, thirty stakeholders contributed during this study. Twenty-two staff members participated over four focus groups, with one focus group occurring in each study location – Broken Bow, NE, Columbus, NE, Lincoln, NE, and Omaha, NE. Table 2 describes the breakdown of the focus group participants by occupation. The Broken Bow focus group had eleven participants, Columbus had eight participants, and Lincoln had two participants. Omaha had an interview instead due to only recruiting one participant. One participant agreed to participate in the Lincoln focus group but did not attend. Eight parents and caregivers participated in the interviews, with 104 participants contacted and one participant lost to follow-up, as shown in Fig. 1. No reasons were given for declining or dropping out. All 8 parents/caregivers of CMC had EIFs provided by Project Austin, and two families had used their EIF in at least one emergency.

### Focus Groups with Emergency Medical Providers

Themes identified from discussion with CMC emergency medical providers are displayed in Table 2. The variety of roles who contributed to discussion produced a diverse collection of themes about the EIFs. Most participants reported being familiar with Project Austin and had experience working with CMC during emergencies. Themes identified during focus groups were condensed into codes addressing either the content of the EIF (Table 3) or the care processes while using the EIF (Table 4).

The information required to care for a CMC represents a good summation of the content of an EIF form, covering their diagnosis, short past medical history, medications, and the baseline for the CMC. Social information is missing from the EIF. The comment that the EIF is handwritten was unexpected and concerning. EIFs from Project Austin were all printed, rather than handwritten, and have not been for some time at the start of the study. Most participants had something to contribute on changes that could be made to the EIF in Table 3, with many of them less having to do with the EIF and more with the systems surrounding it (i.e., facilitate meetings between EMS and CMC, or referring children to Project Austin). Since the EIF has varying requirements across many different users, many commented on wanting changes that would make the EIF more relevant to them such as, limiting of complex medical language, provider-type-specific sheets, and tiered complexity.

The process of using the EIF also seems to vary by both profession and location, as shown in Table 4, as there was no single consistent method of maintaining and searching for the relevant EIF form. The EIF forms were often kept together for all children, making them difficult to access in an emergency. Many EMS services reported are reliant on families to provide the EIF and specialized equipment. The presence of a “go-bag” for emergencies was a topic common to both EMS and parents/caregivers. When the first-line information sources are unavailable to EMS and ED providers, they are either left to hope that the child’s normal care providers can provide an adequate history either verbally or through a note in the EHR, or to treat the child exactly as they present.

**Table 2:** Focus Group Participants

Participant Occupation	Number of Participants
Physicians	6
Physician Assistant	1
Nurses	4
Patient Care Coordinators	4
EMS instructors	2
EMS Managers (Ambulance, Fire Department, Acute Care)	5

**Table 3:** **Focus Group Themes about EIF Content [End of Document].** These quotes were made during one of the four focus groups conducted with ED providers and EMS about the specific content of the EIFs.

Themes Identified	Quotes
**Critical Information in an Emergency for CMC**	
Airway Difficulty	*“… knowing [beforehand] who has the difficult airways…” (Focus Group 4)*
Allergies	*“…knowing any allergies that they have.” (Focus group 3)*
Diagnosis	*“…so having someone spell [the diagnosis] out… is very helpful for me.” (Focus group 1)*
Advanced care directive	*“What their DNR status is.” (Focus group 1)*
Past medical history	*“I’d say their past medical history.“ (Focus group 2)*
Medications	*“I like the drugs that they are on, on there, and the dosage.“ (Focus group 2)*
What labs to draw	*“. Often they have what labs to draw and if there is one that we don’t typically think of to draw.“ (Focus group 2)*
Normal vs. Abnormal	*“… a good understand of what’s normal for the patient versus abnormal.“ (Focus group 4)*
Date of last update	*“… so, keeping that form updated on a fairly regular basis is kind of important. Sometimes you look at it and you think that’s really old, I’m not sure that’s still current.“ (Focus group 2)*
**Skills/Knowledge Needed to Provide Care**	
Confident in current skills	*“I feel very confident that we can handle any situation we come into.“ (Focus group 3)*
Interest in continuous training	*“We always welcome further education.“ (Focus group 3)*
Interested in simulation training with a pediatric focus	*“99% of [simulation training] for us is going to be airway support.“ (Focus group 1)*
Interest in educational videos	*“If you could just get the emergency information and then we could go forward into the educational videos, that would be nice.“ (Focus group 1)*
**Information Missing from EIF**	
No missing information	*“No, I don’t think so. I think it’s set up nicely.“ (Focus group 3)*
Emergency plan for displacement from home	*“…and I actually felt there should be some plan on there for if their home is for some reason gone, flooded, fire, tornado…” (Focus group 1)*
Social information	*“Who is in the home, who has guardianship, who can sign for the child, who is responsible for decisions…” (Focus group 1)*
Changes in last EIF update	*“Things that have updated or changed is probably one of the biggest [issues].“ (Focus group 2)*
No introduction between child and local services	*“When the program started, I thought there was going to be a representative […] setting up a meeting between EMS, momma, and the ED…” (Focus group 1)*
**Issues when Caring for CMC with EIFs**	
Handwritten EIF	*“You know what, a lot of it’s handwritten.“ (Focus group 1)*
EMS responders do not see EIF	*“I think the form probably gets sent to someone and filed away, and then they rely on the parents.“ (Focus group 1)*
Language Barrier	*“…we have different languages other than English…” (Focus group 1)*
Oversaturation of Project Austin	*“It seems like so many kids were getting enrolled in the program that it was becoming oversaturated with lower acuity patients.“ (Focus group 4)*
Medical Terms are difficult to understand	*“…there’s diagnoses that I have no idea what they even are […] the medical terminology is awesome, but sometimes just putting it in basic terms is better.“ (Focus group 4)*
EMS responders do not have access to the EHR	*“Out in the field, if we wouldn’t know, it makes it a lot harder to figure out since we don’t have databases to fall back on or anything like that.“ (Focus group 3)*
Difficulty Performing Procedures	*“We have had a couple incidences we had a hard time getting their port accessed or getting an IV.“ (Focus group 2)*
**Improvement Suggestions**	
Profession Specific Tabs	*“I think an EMS section, like an EMS tab or button would be helpful too.“ (Focus group 1)*
Update when New Child is enrolled or moves	*“So she was saying if we could get an updated list, like every six months or so, staking the kids hat are in the area that are a part of Project Austin, so we know who is still here.“ (Focus group 2)*
Facilitate meeting between family and EMS before emergency	*“I think that a key role is being able to meet with those caregivers ahead of time.“ (Focus group 4)*
Alert when responding to call from Project Austin child	*“It would be nice if, when you are driving to that address, something would pop-up and then you would have [the EIF].“ (Focus group 1)*
Tier-system of Medical Complexity	*“I think if there was a different level tiered system, where we could have those higher acuity patients listed, that would be helpful.“ (Focus group 4)*
Able to refer children to Project Austin	*“To be honest with you, [children with cancer] are the kids we’re seeing constantly, and we have nothing on them.“ (Focus group 2)*

**Table 4:** **Focus Groups Themes on EIF in the Care Process.** These quotes were made during one of the four focus groups conduced with ED providers and EMS about workflows around utilization of the EIF forms and processes around CMC children

Themes Identified	Quotes
**Process of Gathering Information**	
Electronic Health Record	*“…and any past medical record we might have either here or through the clinic.“ (Focus group 2)*
Parents and caregivers	*“We would mostly just go off the parents and caregivers and get all that information from them.“ (Focus group 3)*
School nurse	*“… or the school nurse, if they have any information.“ (Focus group 4)*
**Process if Informational Resources are Unavailable**	
Contact the hospital	*“We could call Children’s and get a report…” (Focus group 1)*
Interview whoever is at the scene	*“… or [ask] somebody that knew the child, we’d have to just ask questions and figure it out.“ (Focus group 4)*
Electronic Health Record from other hospitals	*“We use the online electronic health record server all the time, but sometimes at 2 am it’s hard to find a computer that has the sensitive server.“ (Focus group 1)*
Transfer patient to nearby children’s hospital	*“There are times when we end up sending them to the nearest facility that has pediatricians…” (Focus group 2)*
Treat based on current presentation only	*“Basically, just treat them as whatever, however they present.“ (Focus group 3)*
**Process of Retrieving Information from the EIF**	
No issues	*“No, I don’t think [there are issues].“ (Focus group 2)*
Pulls sheet from binder	*“We have the Project Austin paperwork all in a binder in the ED, so we just have to grab it.“ (Focus group 2)*
Not enough time to look for sheet	*“… we don’t have time to look through the book to find them.“ (Focus group 1)*
Helpful when included in child’s “go bag”	*“… putting the EIF by their go-bags, and I think that’s awesome…” (Focus group 4)*
**Access to Equipment Needed to Provide Care**	
Yes, it is available	*“I believe we carry everything that we need, at least for all the [Project Austin kids] that have come through so far.“ (Focus group 3)*
No, but family provides it	*“We tell them that they have to have their own [equipment] and they have to bring it, and they have to make sure that if I put one in, they have a new one at home.“ (Focus group 1)*
No, the equipment is unique to the child	*“That’s difficult to answer because they are all so individual.“ (Focus group 2)*
No, the equipment expires	*“We used to do that, but they would expire, and then you’d have this big pile of them.“ (Focus group 1)*

### Key informant interviews with Parents/Caregivers

Themes identified from discussions with parents and caregivers is displayed in Table 5. All interview participants were part of Project Austin and currently had an EIF for one or more of their children. Parents and caregivers consistently can state the goals of the Project Austin initiative and the purpose of the EIF for their child. Most of the form was completed by the staff of Project Austin with the assistance of parents and caregivers. Many agreed that the form helped them maintain the complicated information for their children. All parents entered Project Austin either through being referred to the Complex Care Clinic or receiving a pamphlet for Project Austin. It appears that parents/caregivers take on the role of verifying and maintaining the EIF. Of the parents and caregivers interviewed, only 2 reported a medical emergency requiring them to call EMS or go to the ED. Of these situations, one reported an improved care experience, and the other reported a variable care experience over multiple ED visits. The interviewee with variable care reported that staff familiarity with the EIF seemed to be at fault, not the actual form itself.

After the interview, many of the participants asked follow up questions about a possible conversion to an electronic management system. They expressed concerns about current hospital access to technology, if it will be effective and accessible, and how they will identify their child as part of Project Austin. One mentioned a desire to have state-level access to the EIF.

**Table 5:** **Key Informant Interview Themes.** These quotes were provided during one of the eight key informant interviews with parents and caregivers for CMC.

Themes Identified	Quotes
**Benefits from being a part of Project Austin**	
Far from specialty care clinic	“*Due to the fact we live in a more rural community, and there aren’t many patients like my son.”* (Interview #2)
Easy access to critical information	“*Just so he can get the care that he needs… faster?… I don’t have to have everything on hand.*” (Interview #6)
EIF is used	*“Yes, they [EMS] pull it every time we’re in there.” (Interview #7)*
**The process of creating the EIF**	
Referred to Project Austin	*“…I can’t remember who referred me, but they gave me a pamphlet.*” (Interview #7)
Filled out the EIF	“*They gave me a [EIF] template and I just called the phone number… and I talked to a lady there*.” (Interview #7)
**Parental/Caregiver role in the EIF creation process**	
Assisted Project Austin team in filling out EIF	*“… they [Project Austin Staff] just came in and asked us several questions about her health history… and then they printed out the emergency form for us*.” (Interview #8)
Ensures EIF is complete and correct	“*I’m just like, basically, confirming information, going over stuff like medication he’s on and confirming his diagnosis.”* (Interview #6)
Identify their child as part of Project Austin	*“…the easiest part was just telling them [EMS] she was a Project Austin kiddo.*” (Interview #1)
Annual updates to EIF	*“About once a year, I will get in contact with somebody from Project Austin and we go over the sheet we already have and make any updates as necessary.” (Interview #1)*
**Parental/Caregiver goal with having an EIF**	
Emergency Medical Services better prepared	“*Just ensuring that if there is ever an emergent need that they kind of know what the care plan should be for her*.” (Interview #5)
**Positive effects of EIF**	
Project Austin improved care	*“… Ever since we’ve been a part of Project Austin, things have been going really smoothly. Before Project Austin it was kind of a cluster*.” (Interview #7)
**Improvable aspects of EIF**	
Perceived Lack of Training	“*So, there really isn’t anything that has gone super great with our local ER, and mainly because they aren’t well-versed with it…”* (Interview #1)
Unsure if EIF is Used	“*I think they do that [look at the form]*?” (Interview #1)
Treats Calls as Trivial	“*Just, them [EMS] taking it seriously when I call them and tell them she is a project Austin kiddo*.” (Interview #1)

Our study of parents and emergency medical providers revealed that respondents found the EIF to be an easy way to engage parents, caregivers, and emergency personnel about the specifics of a CMC’s care before or during an emergency. They also identified areas for improvement regarding the organization, deployment, and optimal usage of the EIF in practice. EIFs are designed to be used by emergency medical providers both in EDs and in the field, but respondents reported that EIFs are not ideally adapted for either purpose. For example, the EIF, as a physical document, must be securely stored by ambulances and families, yet also be physically available for immediate use in the field and/or once the child reaches the ED. Separate copies stored by emergency medical providers and families results in patient safety threats, as one or both copies may no longer be up to date. These barriers can decrease the utility of the EIF, particularly when a provider does not feel that the information is trustworthy.

These results are consistent with previous published literature that highlights the need for trustworthy medical information on CMCs during medical emergencies. Pulcini et al. (2021) found that parents/caregivers of CMC believed that insufficient data exchange occurred between parents and physicians during medical emergencies, especially when communicating complicated medical history [18]. Pediatric emergency medicine providers identified similar challenges, and many providers suggested that electronically delivered management recommendations (rather than scouring the electronic health record manually) could optimize emergency care. Our study expands on these insights by interviewing non-physician healthcare providers, who highlighted similar concerns. Larson et al. (2020) similarly found that while the usage of the EIF did not necessarily improve the comfort or preparation of providers, it did improve the provider’s self-perceived communication during an emergency [19].

Our work highlights a previously identified opportunity to convert EIFs to an electronically accessible version. Many of the concerns previously identified would be eliminated by the implementation of an electronic EIF. In the 2010 AAP policy statement on EIFs and Emergency Preparedness for Children with Special Healthcare Needs, the utilization of physical EIFs are limited due to lack of awareness, are sub-optimal for incorporation into electronic medical records, and are [7]. Findings from our study support the recommended content for EIFs outlined in the AAP policy statement, including information on patient allergies, medications, diagnoses, presence/absence of advanced care directives, and overall severity/risk of clinical deterioration. Electronic EIFs could enhance the safety of this vital information through automatic updates of the form using information sources housed within electronic health records. Such updates could address some of the information trustworthiness concerns expressed by our participants regarding paper EIFs. Copper et al. (2020) interviewed a mixed group of stakeholders, including parents, healthcare providers, health information technologists, and privacy compliance experts, and found similar content recommendations for a web-based, easily updated EIF maintained by providers [20].

Project Austin is unique for its focus on the specific barriers that rural families with CMC experience and for attempting to meet that need through targeted emergency medical provider training and education. Use of the paper EIF has been a central element of Project Austin since its inception. Based on responses from our participants, future work on the EIF should focus on increasing trust in the EIF’s content being up to date, accurate, and readily available. An electronic EIF that prioritizes at-a-glance necessary information for a variety of emergency medical providers that is current and accurate may address many of the concerns expressed by parents and emergency medical providers in our study. Continued stakeholder engagement will be key in developing such a product.

### Limitations

This study had limitations. First, data was collected over the start of the SARS-CoV-2 pandemic, and this affected focus group attendance and could have affected participation in the key informant interviews. Second, the personal biases of the investigators could have affected interview interpretation. It was disclosed to participants that the future goals of this research were to convert the EIF from a paper to electronic form, which could have influenced the participants’ follow-up questions. Third, the small sample size of the focus groups and key informant interviews may limit the generalizability of our team’s findings. We also did not request any specific information about the CMCs, including medical information or about the providers included in the study. This could limit the generalizability of these findings. Lastly, many of the participants had never activated their EIF through Project Austin, and the answers were hypotheticals based on their opinions.

## Conclusions

The EIF is an easy way to engage parents, caregivers, and emergency medical personnel about the care specifics for CMC during an emergency, even if, when activated, the results are reportedly mixed. Timely updates and electronic access to EIFs could improve their value to parents, caregivers, and emergency medical providers.

## Electronic supplementary material

Below is the link to the electronic supplementary material.

Supplementary Material 1

## Data Availability

The datasets generated and/or analyzed during the current study are not publicly available due to interviews containing possibly identifying information, but the codebook is available from the corresponding author on reasonable request.
