# Supplementary material for: An assessment of information needs and workflows for emergency service providers and caregivers of children with medical complexity
Source: BMC Health Serv Res. 2023 May 8;23:453. doi: 10.1186/s12913-023-09366-y (PMC10166030; doi:10.1186/s12913-023-09366-y)
Supplement: Supplementary file 1 — Supplementary Material 1 [file 12913_2023_9366_MOESM1_ESM.docx]

Interview:

Probing questions

1.     Can you say more about that?

2.     Can you be more specific?

3.     Can you give me an example of that?

4.     Do others have similar or different experiences to share?

Laddering questions

1.     “Which feature do you like best?”

2.     “What is important about that?”

3.     “What does that do for you?”

4.     “What does the benefit do for you?”

Is your child a part of Project Austin?

Does your child have an Emergency Information Form (EIF)?

*Project Austin group*

How does your child benefit from being a part of Project Austin?

Describe to me the process of creating the EIF for your child?

What was your role in the creation process?

What is your goal with having the EIF for your child?

Have you had to activate Project Austin and access the EIF in an emergency situation?

If so, describe what went well with the use of the EIF?

Describe what could have gone better?

*Not in Project Austin*

Do you have concerns for your child’s care in the event of an emergency?

What specific care items concern you the most?

Has your child had to be treated by EMS or an ED?

How were your child’s specific care needs communicated with care providers?

What aspects of care went well in that emergency situation?

Describe what could have gone better?

Focus Groups:

What information is critical to providing emergency care to medically complex kids?

How do you typically gather this information?

If you can’t get the information, are you able to adapt your care processes?

If you get the information, do you have the equipment to care for the child?

Do you have the skills/knowledge to care for the child?

Have you treated a project Austin patient?

Describe the process of retrieving information from the EIF?

Was information missing?

Did you receive the information in a timely manner?

Were you able to change the way you provided care based on the information?
